# Supplementary material for: In Vitro Evaluation of the Therapeutic Potential of Phage VA7 against Enterotoxigenic Bacteroides fragilis Infection
Source: Viruses. 2021 Oct 11;13(10):2044. doi: 10.3390/v13102044 (PMC8538522; doi:10.3390/v13102044)
Supplement: Supplementary file 1 [file viruses-13-02044-s001.zip › Figure S1.pdf]

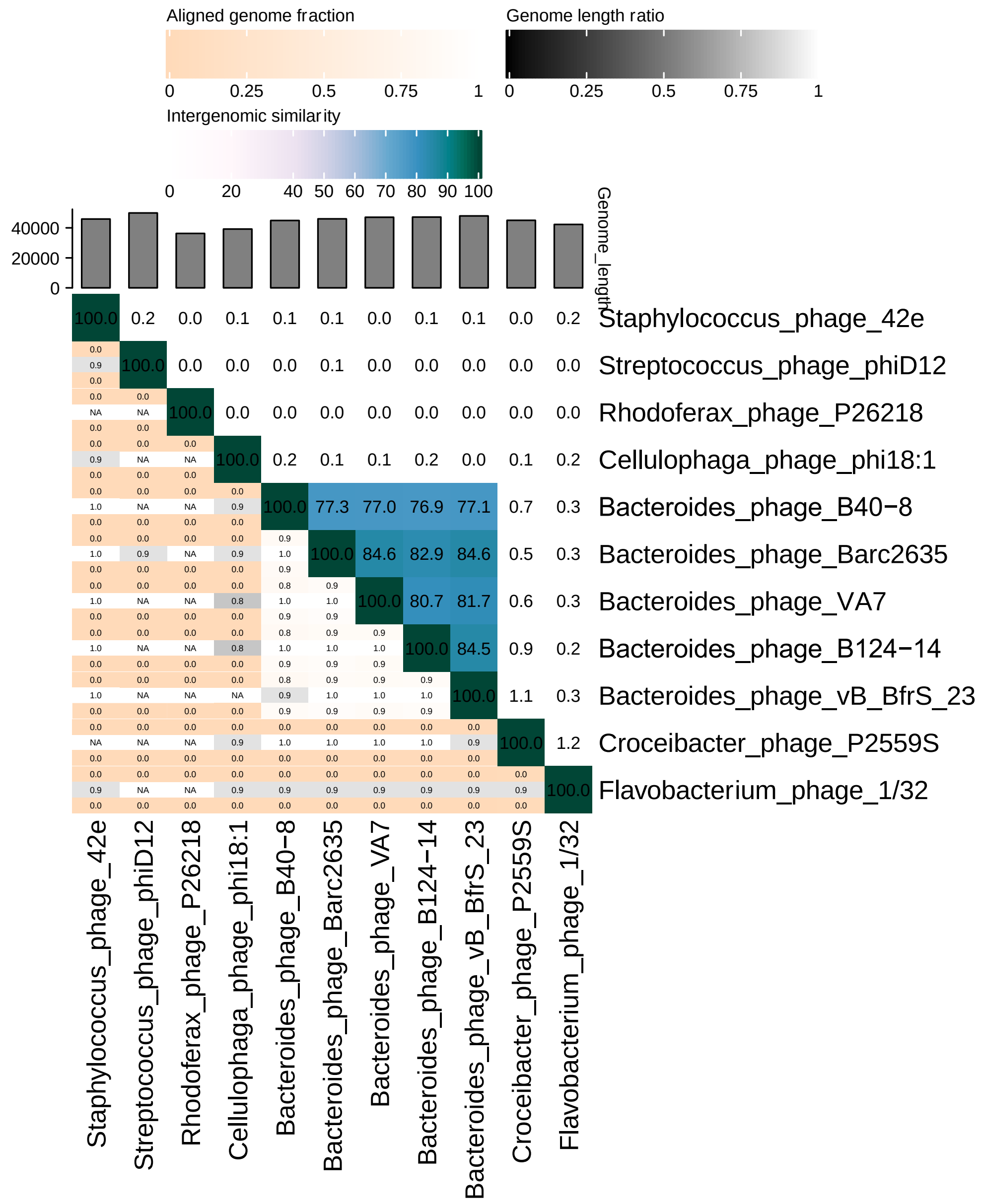

Figure S1. VIRIDIC (Virus Intergenomic Distance Calculator) heatmap comparing the phages most related to VA7. The legend shows the intergenomic similarity between each pair of two phages as a percentage of sequence identity. Phages sharing more than 70% identity are members of the same genus, thus showing VA7 belongs to the same genus as e.g., B124-14.
