# Supplementary material for: In Vitro Evaluation of the Therapeutic Potential of Phage VA7 against Enterotoxigenic Bacteroides fragilis Infection
Source: Viruses. 2021 Oct 11;13(10):2044. doi: 10.3390/v13102044 (PMC8538522; doi:10.3390/v13102044)
Supplement: Supplementary file 1 [file viruses-13-02044-s001.zip › Figure S2.pdf]

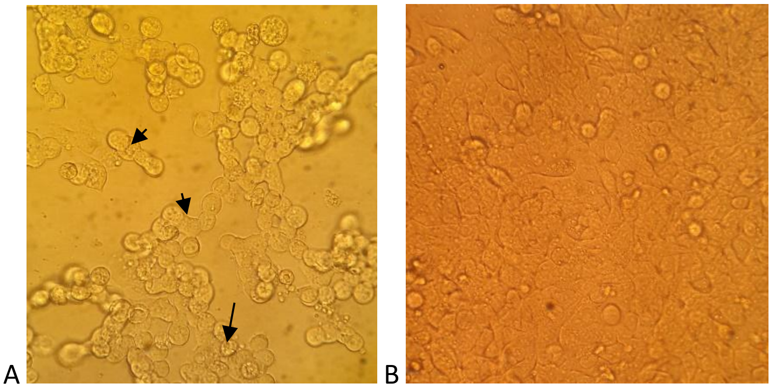

Figure S2. Cytotoxicity assay using colon cancer cells (CECs), adapted from Pantosi *et al.* Inverted microscope images of the cytotoxicity assay: (A) HCT116 CECs, after incubation during 2-4 h with the supernatant of enterotoxigenic *Bacteroides fragilis* strain E3. Arrows point at the rounded and detached epithelial cells (B) Unaffected spindle shaped, tightly joined HCT116 CECs after incubation with the supernatant of the nontoxigenic *B. fragilis* strain ATCC 25285^T. Magnification x 250. Pantosti, A.; Cerquetti, M.; Colangeli, R.; D'Ambrosio, F. Detection of intestinal and extra-intestinal strains of enterotoxigenic Bacteroides fragilis by the HT-29 cytotoxicity assay. *J. Med. Microbiol.* **1994**, *41*, 191-196.
